# Guilty or Not Guilty: Does Venovenous Extracorporeal Membrane Oxygenation Directly Harm the Kidney?

**DOI:** 10.34067/KID.0000001093

**Published:** 2026-01-29

**Authors:** Luis A. Juncos, Thiago Reis

**Affiliations:** 1 Medical Affairs, Fresenius Medical Care, Waltham, Massachusetts; 2 Division of Nephrology, University of Arkansas for Medical Sciences, Little Rock, Arkansas; 3 Division of Nephrology, University of São Paulo School of Medicine, São Paulo, Brazil; 4 CPQuali Pesquisa Clínica, Clinical Research Center, São Paulo, Brazil; 5 Hospital Beneficência Portuguesa, São Paulo, Brazil

**Keywords:** acute kidney failure, AKI, risk factors

Extracorporeal membrane oxygenation (ECMO) has evolved from an experimental salvage therapy to an established treatment for severe respiratory or cardiac failure unresponsive to conventional care.^1,2^ Two configurations exist: venoarterial (VA) ECMO, in which venous blood is withdrawn, oxygenated, and returned to the arterial circulation to provide both circulatory and respiratory support, and venovenous (VV) ECMO, in which oxygenated blood is returned to the venous system, enabling gas exchange while relying on native cardiac function for systemic perfusion.^3^ These fundamental distinctions create distinct physiologic differences, with VA-ECMO affecting afterload and pulsatility, whereas VV-ECMO primarily affects venous return and right-sided pressures. Both modalities expose blood to artificial surfaces, require anticoagulation, and alter hemodynamics and inflammatory responses. Despite major technical and clinical advances, multiorgan dysfunction remains common during ECMO, with AKI occurring in 40%–70% of patients.^4–6^

**Figure 1:**
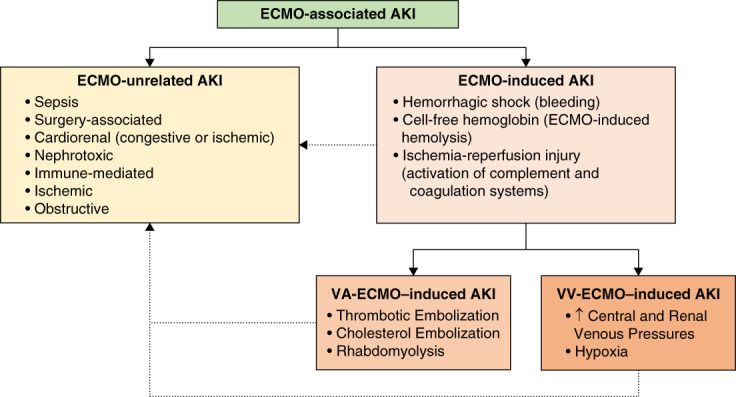
**ECMO-associated AKI.** In patients receiving ECMO, the etiology of AKI is multifactorial. The underlying condition leading to respiratory and/or cardiac failure that ultimately leads to ECMO initiation may also contribute to kidney damage. Because establishing a causal relationship between ECMO and AKI can be challenging, the term ECMO-associated is more appropriate. Some causes of AKI, such as urinary tract obstructive complications, are unrelated to the ECMO procedure *per se*. Nonetheless, factors intrinsic to ECMO can exacerbate or perpetuate AKI that originated independently of ECMO. Furthermore, the mechanisms by which ECMO contributes to AKI include some that are shared by both VA and VV configurations, and others that are specific to each. ECMO, extracorporeal membrane oxygenation; VA, venoarterial; VV, venovenous.

Most previous work on ECMO-associated AKI has focused on the VA configuration, whereas less is known about AKI in VV-ECMO.^4,5^ This knowledge gap is important because VV-ECMO use has expanded rapidly, particularly as a bridge in severe acute respiratory distress syndrome. Although VV-ECMO is considered less invasive with fewer hemodynamic disturbances, available data do not show a lower risk of AKI: it still occurs in 50%–75% of patients receiving VV-ECMO, of whom 40%–50% require KRT.^6^ The persistence of such high rates of AKI despite the physiologic differences suggests that the fundamental mechanisms of renal injury—both ECMO dependent and independent—remain operative regardless of circuit configuration. These mechanisms include perturbations in renal perfusion, systemic inflammation, activation of complement and coagulation cascades, hemolysis with pigment exposure, venous congestion, and nephrotoxin exposure (Figure 1).^7^ Thus, although the physiologic profiles of VA and VV support differ substantially, the underlying pathways of AKI appear largely shared, and the extent to which they are influenced by circuit design remains uncertain.

Against this background, the study by Fuchs *et al*.^8^ in this issue provides valuable data addressing this gap. The authors examined the incidence, timing, and outcomes of AKI in 500 consecutive patients supported with VV-ECMO at a single center between 2014 and 2021. Using Kidney Disease Improving Global Outcomes creatinine criteria, AKI was categorized according to its temporal relationship with ECMO initiation—before and during ECMO or new AKI during ECMO after an initial recovery—and patients were followed for 1 year after hospital discharge. The authors used propensity-score matching to compare patients who developed AKI during ECMO with those who did not. AKI occurred in 64% of patients, and 44% required KRT. The median time to AKI onset during ECMO was 3 days. Hospital survival was 67%, and 1-year survival among hospital survivors was 92%. In multivariable analysis, only stage 3 AKI independently predicted in-hospital mortality, regardless of timing. As a benchmark, in a 2020 international Extracorporeal Life Support Organization registry of 4812 patients who received ECMO during the coronavirus disease 2019 pandemic, in-hospital mortality varied from 37% during the first wave up to 52% thereafter.^9^ In that cohort, AKI was associated with mortality, along with advanced age, cancer, and cardiac arrest before ECMO.

In their analysis, the authors also found that patients who developed AKI during ECMO had lower arterial pressures, and higher C-reactive protein concentrations preceded diagnosis, whereas ECMO flow rates, cell-free hemoglobin, and ventilator parameters were similar between groups. By contrast, in a retrospective analysis by Pilarczyk *et al*.,^6^ initial ECMO blood flow was higher in patients who developed AKI.^10^ However, that study compared patients with AKI stage 2 or 3 to those with no AKI or AKI stage 1 and excluded individuals with preexisting AKI or AKI that recovered before ECMO initiation, making cross-study comparisons difficult and potentially misleading.

This study advances our understanding of AKI in VV-ECMO by confirming that severe AKI is common, temporally variable, and strongly associated with mortality, while also incorporating the timing of injury relative to the course of support and highlighting that underlying mechanisms remain incompletely understood. Its association with hypotension and elevated inflammatory markers suggest that AKI largely reflects the broader physiologic dysregulation of critical illness rather than processes intrinsic to the ECMO circuit. Nonetheless, these results should be interpreted cautiously because the retrospective design limits causal inference and interactions between systemic and circuit-related factors cannot be excluded and are likely interdependent.

The study's main strength lies in its detailed phase-based characterization and large, well-defined cohort, enabling comparisons across different phases of VV-ECMO. The use of consistent institutional practices and standardized Kidney Disease Improving Global Outcomes criteria lends internal validity and allows alignment with other studies. Propensity matching between patients with and without ECMO-associated AKI enhances analytical rigor and reduces confounding. Importantly, no ECMO-specific variable, such as circuit flow, sweep gas rate, or cell-free hemoglobin, independently predicted AKI. This finding supports the interpretation that intrinsic host factors—hemodynamic derangements, inflammation, and metabolic stress—likely play a greater role than circuit characteristics in the development of AKI.

Nonetheless, several limitations temper the conclusions. The retrospective, single-center design limits generalizability, and data completeness was affected by the introduction of a new patient data management system midway through the study. AKI was defined solely by changes in serum creatinine without urine output, perhaps leading to underestimation. Renal recovery or long-term kidney outcomes were not assessed, leaving uncertainty about reversibility or progression. Associations between mean arterial pressure, inflammatory markers, and subsequent AKI should be interpreted cautiously because these variables are surrogate indicators of systemic stress rather than specific mechanistic drivers. The conclusion that recurrent sepsis was a principal cause of renal injury is plausible but unproven because microbiologic and cytokine data were not collected. Likewise, the lack of association between ECMO flow or hemolysis parameters and AKI does not exclude circuit contributions; subtle microcirculatory or oxygen delivery abnormalities may have escaped detection with available metrics.

In sum, the study by Fuchs *et al*.^8^ advances our understanding of AKI in VV-ECMO while underscoring that much remains unresolved. The persistence of high AKI rates despite advances in technology suggests that AKI reflects the broader physiology of critical illness, with potential contributions from extracorporeal therapy. The absence of clear modality-specific predictors does not exclude a role from circuit-related factors but indicates that they are difficult to isolate from the systemic milieu in which ECMO operates. The consistent association between severe AKI and mortality underscores its clinical importance, but without mechanistic clarity, treatment remains largely supportive. Optimizing volume status and hemodynamics, while avoiding nephrotoxins, is necessary but insufficient because these measures may not address the main underlying injury process. A next step is to classify AKI according to its temporal relationship with ECMO—before initiation, during initiation, during therapy, and after liberation—linking physiologic observations to clinical context to determine which injury pathways predominate at each stage. Progress may require obtaining more granular physiologic and molecular data—such as improved hemodynamic monitoring, assessment of renal oxygenation, and molecular biomarkers of endothelial and inflammatory activation—rather than reliance on traditional clinical end points. The work by Fuchs *et al*.^8^ is, therefore, both informative and a reminder of the limits of our knowledge: it advances the characterization of AKI in VV-ECMO while emphasizing that moving from association to causation remains essential if future research is to yield strategies that prevent or mitigate AKI in this increasingly common form of life support.
